# High Adherence to Pharmacological Treatment Guidelines in Recovery and Remission States Among Patients With Schizophrenia: A Cross‐Sectional Study

**DOI:** 10.1002/npr2.70136

**Published:** 2026-06-10

**Authors:** Ryo Asada, Hitoshi Iida, Leo Gotoh, Kiyohiro Yasumatsu, Hikaru Hori

**Affiliations:** ^1^ Department of Psychiatry, Faculty of Medicine Fukuoka University Fukuoka Japan; ^2^ Laboratory of Neuroscience, Department of Psychiatry, Faculty of Medicine Fukuoka University Fukuoka Japan

**Keywords:** guideline, non‐remission, pharmacotherapy, recovery, remission, schizophrenia

## Abstract

**Background:**

Several schizophrenia guidelines have been published, showing treatment efficacy. However, no studies have examined recovery and pharmacological therapy or guideline adherence's impact on various schizophrenia states. This study aimed to investigate adherence to pharmacological guidelines across recovery, remission, and non‐remission states in patients with schizophrenia.

**Methods:**

This cross‐sectional study included 72 patients with schizophrenia who met the criteria for recovery, remission, or non‐remission. Adherence to pharmacological guidelines was measured using the Individual Fitness Score (IFS).

**Results:**

IFS was significantly higher in the recovery (88.1 ± 18.9) and remission (89.0 ± 16.7) groups than in the non‐remission group (65.59 ± 21.8) (*p* < 0.01). However, no significant differences were observed between the recovery and remission groups. Receiver operating characteristic analysis identified 72 points on the IFS as a potential cutoff point between remission and non‐remission.

**Conclusion:**

These results show that higher adherence to pharmacological guidelines may be associated with recovery and remission state, compared with non‐remission state. The cutoff point between remission and non‐remission should be considered preliminary and exploratory because of a small sample. Clinical practice guidelines have been developed to standardize and improve the quality of medical care. Treatments following these guidelines have been reported to be effective.

## Abstract 2

Guideline‐adherent pharmacotherapy (Individual Fitness Score: IFS) was higher in recovery and remission than in non‐remission in schizophrenia (≈88–89 vs. 66; *p* < 0.01). An IFS cutoff of 72 discriminated remission from non‐remission. Findings suggest adherence may support favorable clinical states, though the cutoff is preliminary (small sample, cross‐sectional).

## Graphical abstract

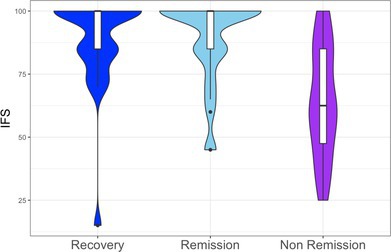

## 1 Background

Several guidelines for schizophrenia have been published, and treatments based on these guidelines and algorithms have been shown to be effective in patients with schizophrenia [1, 2]. In schizophrenia pharmacotherapy, many guidelines recommend antipsychotic monotherapy to minimize the side effects of antipsychotics or other psychotropics [3, 4, 5]. In Japan, pharmacological therapy guidelines for schizophrenia have been published by the Japanese Society of Neuropsychopharmacology (JSNP) [5]. These guidelines recommend second‐generation antipsychotic (SGA) monotherapy without the combination of other psychotropic drugs for acute and chronic patients with schizophrenia. The individual fitness score (IFS) is a new tool used to evaluate schizophrenia treatment adherence to guidelines [6] and assess compliance with the JSNP guidelines. Previous studies using the IFS to evaluate adherence to these guidelines showed that high adherence may contribute to improved psychiatric symptoms [7] and working time [8].

Recovery after achieving remission is considered one of the goals of patients with schizophrenia. Recovery involves improving symptoms, social function, and personal relationships [9]. However, a systematic review reported a recovery rate of 13.5% [10], which has remained low compared with the remission rate [11]. Furthermore, to the best of our knowledge, no studies have examined the relationship between recovery in schizophrenia and pharmacological therapy. In addition, previous studies using the IFS evaluated treatment‐resistant schizophrenia (TRS) and non‐TRS [7, 8]; however, no evidence has demonstrated that adherence to guidelines contributes to several states of schizophrenia, including recovery or remission.

Thus, adherence to the guidelines may improve psychiatric symptoms, including hallucinations or delusions. However, it remains unclear whether adherence to the guidelines contributes to achieving recovery or remission in patients with schizophrenia. We hypothesized that the IFS would differ depending on the state, such as recovery, remission, and non‐remission, in patients with schizophrenia, and some points on the IFS could be indicators to differentiate between these groups. Therefore, this study aimed to compare the IFS across recovery, remission, and non‐remission patients with schizophrenia. The secondary purpose was to evaluate the cutoff points for each group.

## 2 Methods

### 2.1 Participants

This cross‐sectional study recruited participants from five psychiatric institutions (Fukuoka University Hospital, Aburayama Hospital, Rainbow and Sea Hospital, Kawano Clinic, and UNB Sumiyoshi Jinja Mae Clinic in Japan) between 2021 and 2024. In total, 72 Japanese patients with schizophrenia were enrolled in this study. The inclusion criteria were as follows: (1) diagnosis of schizophrenia based on the Diagnostic and Statistical Manual of Mental Disorders, Fifth Edition (DSM‐5) criteria [12], (2) age 20–65 years; (3) no change in the dose of antipsychotics for ≥ 3 months; (4) not taking anticholinergic drugs; and (5) willingness to provide written informed consent. The exclusion criteria were as follows: (1) history of trauma with loss of consciousness or serious organic disease, including central nervous system diseases, and (2) diagnosis of substance‐related and addictive disorders based on the DSM‐5 [12]. We collected clinical (medication prescriptions, duration of illness, age of onset, and number of admissions) and demographic data (age, sex, body mass index (BMI), marital status, smoking, and duration of education or employment) from medical records and interviewed the participants.

Clinical trial number: not applicable.

### 2.2 Definition of Recovery, Remission, and Non‐Remission

The definition of recovery in patients with schizophrenia is based on established criteria [9]. This criterion consisted of the following components: (1) symptoms: score ≤ 4 on the positive and negative symptoms assessed by the Brief Psychiatric Rating Scale (BPRS) [13]; (2) vocational function: successful employment in a competitive job or attending school at least half‐time; (3) independent living: living independently without daily supervision; (4) peer relationships: engaging in social interactions with people outside the family at least once per week, maintained for ≥ 2 years.

Symptomatic remission was defined by the following criteria [14]: a score ≤ 3 on the items associated with positive symptoms (items 8, 11, and 15), disorganization (items 4 and 7), and negative symptoms (item 16) on the BPRS, maintained for ≥ 6 months. As the cross‐sectional design, the examiner determined whether requisite conditions for symptom abatement had been met based on the medical records.

There are no established non‐remission criteria, and we proposed the following definition in this study. Non‐remission was defined as not meeting the recovery, remission, or TRS criteria. TRS criteria were defined as not improving a Global Functioning Assessment (GAF) ≥ 41 despite the use of ≥ two antipsychotics (Chlorpromazine (CPZ) equivalent dose of 600 mg or more) for at least 4 weeks. The reason for distinguishing non‐remission from TRS was that the calculation method of IFS differed between TRS and not TRS.

Participants were categorized into three groups using the following procedure: Participants who met the recovery criteria were defined as the recovery group. Patients who did not meet the recovery criteria and met the remission criteria were assigned to the remission group. Those who did not meet the remission and TRS criteria were included in the non‐remission group.

### 2.3 Evaluation of Adherence to the Guidelines

The IFS (see Table 1 by [6]) was used to evaluate adherence to the guidelines. The IFS calculations differed for non‐TRS and TRS; in this study, we used the non‐TRS version. A prescription of SGA monotherapy below the maximum therapeutic dose (MTD) was scored 100 points without deduction. If SGA monotherapy exceeded the appropriate dose, the IFS was calculated as follows: 25 points were deducted for doses within 1.5 × MTD and 50 points for doses exceeding 1.5 MTD. For SGA polypharmacy involving two agents, the IFS was calculated as follows: 25 points were deducted for CPZ equivalents ≤ 1000, 35 points for CPZ equivalents > 1000 and ≤ 2000, and 50 points for CPZ equivalent > 2000. For SGA polypharmacy involving ≥ three agents, 65 points were deducted. Regarding first‐generation antipsychotic (FGA) monotherapy, the IFS was calculated as follows: 5 points were deducted within the MTD, 30 points for doses exceeding the MTD within 1.5 × MTD, and 55 points for doses exceeding 1.5 × MTD. For FGA polypharmacy involving two agents, the IFS was calculated as follows: 35 points were deducted for CPZ equivalents ≤ 1000, 45 points for CPZ equivalents > 1000 ≤ 2000, and 60 points for CPZ equivalents > 2000. Regarding SGA‐ FGA combination therapy, the IFS was calculated as follows: 30 points were deducted for CPZ equivalents ≤ 1000, 40 points for CPZ equivalents > 1000 ≤ 2000, and 55 points for CPZ equivalents > 2000. For SGA and FGA polypharmacy involving ≥ three agents, 70 points were deducted. For concomitant prescription of antidepressants, anxiolytic hypnotics, mood stabilizers, antiepileptic drugs, and other psychotropic drugs (except dopaminergic and anticholinergic drugs), the IFS was calculated as follows: 15 points were deducted for one drug, 35 points for two drugs, and 55 points for ≥ three drugs. For concomitant medication with dopaminergic drugs (dopaminergic anti‐parkinsonian drugs and psychostimulants), 80 points were deducted per drug. The IFS ranged from 0 to 100, with higher scores indicating greater adherence to the guidelines.

**TABLE 1:** Demographics, clinical characteristics and IFS among each schizophrenia group.

	Recovery	SD	Remission	SD	Non‐Remission	SD	*p*	Post hoc
Sex (male/female)	11/13		11/19		11/7		0.258	
Age (year)	43.3	10.3	39.1	10.9	44.8	13.0	0.193	
BMI	23.6	3.9	26.1	5.0	25.3	4.3	0.619	
Education (year)	14.1	1.8	13.6	2.4	13.9	2.3	0.619	
Duration of employment (year)	14.5	9.7	6.1	7.1	5.4	7.1	< 0.001	REC > REM > NREM
Smoking (yes/no)	4/20		4/25		6/12		0.321	
Marital status (single/married/divorced)	14/9 / 1		23/4 / 3		15/2 / 1		0.160	
Duration of illness (year)	17.2	8.9	11.5	7.9	15.8	11.3	0.003	NREM > REM
Average daily dosage (CPZ‐equivalent, mg/day)	355.0	196.7	405.5	174.3	543.2	223.5	0.003	NREM > REC
Number of admissions	1.8	1.2	1.1	0.7	4.1	3.1	0.001	NREM > REM
Age at onset (year)	26.0	7.4	27.7	9.5	23.3	6.0	0.369	
GAF	73.2	5.8	59.9	7.0	51.3	6.7	< 0.001	REC > REM > NREM
BPRS	29.4	6.2	30.0	5.3	43.4	8.0	< 0.001	NREM > REC, REM
CDSS	1.1	1.2	2.9	3.0	4.6	3.7	0.005	NREM > REC, REM
IFS	88.1	18.9	89.0	16.7	65.9	21.8	< 0.001	REC, REM > NREM

Abbreviations: BMI, body Mass Index; BPRS, Brief Psychiatric Rating Scale; CDSS, Calgary Depression Scale for Schizophrenia; CPZ‐ equivalent, Chlorpromazine equivalent; GAF, Global Functioning Assessment; IFS, Individual fitness score; NREM, Non‐remission; REC, Recovery; REM, Remission.

### 2.4 Psychiatric Symptoms

Psychiatric symptoms were assessed using the BPRS and Calgary Depression Scale for Schizophrenia (CDSS) [15]. The BPRS comprises 18 items, each scored on a scale from 1 (not present) to 7 (most severe), with a total score ranging from 18 to 126. Higher scores indicate severe psychiatric symptoms. The CDSS, which consists of nine items, was used to assess depressive symptoms in schizophrenia. Each scale ranged from 0 to 3, with higher scores indicating more severe depressive symptoms.

### 2.5 Evaluation of Functions

The GAF has been widely used to evaluate symptoms and social functioning in psychiatric illnesses [16]. The score ranged from 0 to 100, with a high score suggesting milder symptoms and higher functioning.

### 2.6 Statistical Analysis

The recovery, remission, and non‐remission groups included 24, 30, and 18 patients, respectively. The Statistical Package for the Social Sciences version 27.0 was used for data analysis. The chi‐square test was used for categorical variables, including sex, smoking, and marital status. The Shapiro–Wilk test was used to analyze normality. Age, BMI, and BPRS followed a normal distribution and were analyzed using a one‐way analysis of variance. Education, duration of employment, duration of illness, average daily dose, number of admissions, age at onset, GAF, CDSS, and IFS did not follow a normal distribution and were analyzed using the Kruskal–Wallis test. The Bonferroni correction was used for post hoc comparisons. Spearman's correlation was used to compare the relationship between IFS and clinical characteristics. The IFS cutoff points between the remission and non‐remission groups were determined using ROC curve analysis. Statistical significance was set at *p* < 0.05.

## 3 Results

### 3.1 Demographic Data

The clinical characteristics and demographics of each group are shown in Table 1. Significant differences were observed in duration of employment, duration of illness, average daily dose, and number of admissions between the groups. The duration of illness was significantly higher in the non‐remission group than in the remission group; however, the duration of illness was not significantly different between the recovery and remission or non‐remission groups. The average daily dose in the non‐remission group was significantly higher than that in the recovery group; however, no significant difference was observed between the recovery and remission or remission and non‐remission groups. The BPRS and CDSS scores of the non‐remission group were significantly higher than those of the recovery and remission groups. However, no significant difference was observed between the recovery and remission groups. The GAF score in the recovery group was significantly higher than that in the remission group and significantly higher in the remission group than in the non‐remission group.

Table 2 shows the concomitant use of psychotropic drugs in each group. In the non‐remission group, the frequency of concomitant use of psychotropic drugs tended to be higher, compared to the recovery or remission group.

**TABLE 2:** Details of concomitant use of psychotropic drugs in each group.

Number of subjects taking each medication	Recovery		Remission		Non‐Remission	
	*N*	%	*N*	%	*N*	%
Antipsychotic polypharmacy	3	12.5	3	10.0	6	33.3
Concomitant use of antidepressants	1	4.2	2	6.7	5	27.8
Concomitant use of mood stabilizers	0	0.0	1	3.3	4	22.2
Concomitant use of anxiolytics	5	20.8	1	3.3	4	22.2
Concomitant use of hypnotics	4	16.7	6	20.0	11	61.1
Concomitant use of antiepileptic drugs	0	0.0	1	3.3	0	0.0
Concomitant use of other psychotropic drugs	0	0.0	1	3.3	0	0.0

### 3.2 Comparison of IFS Between Three Groups

**FIGURE 1:**
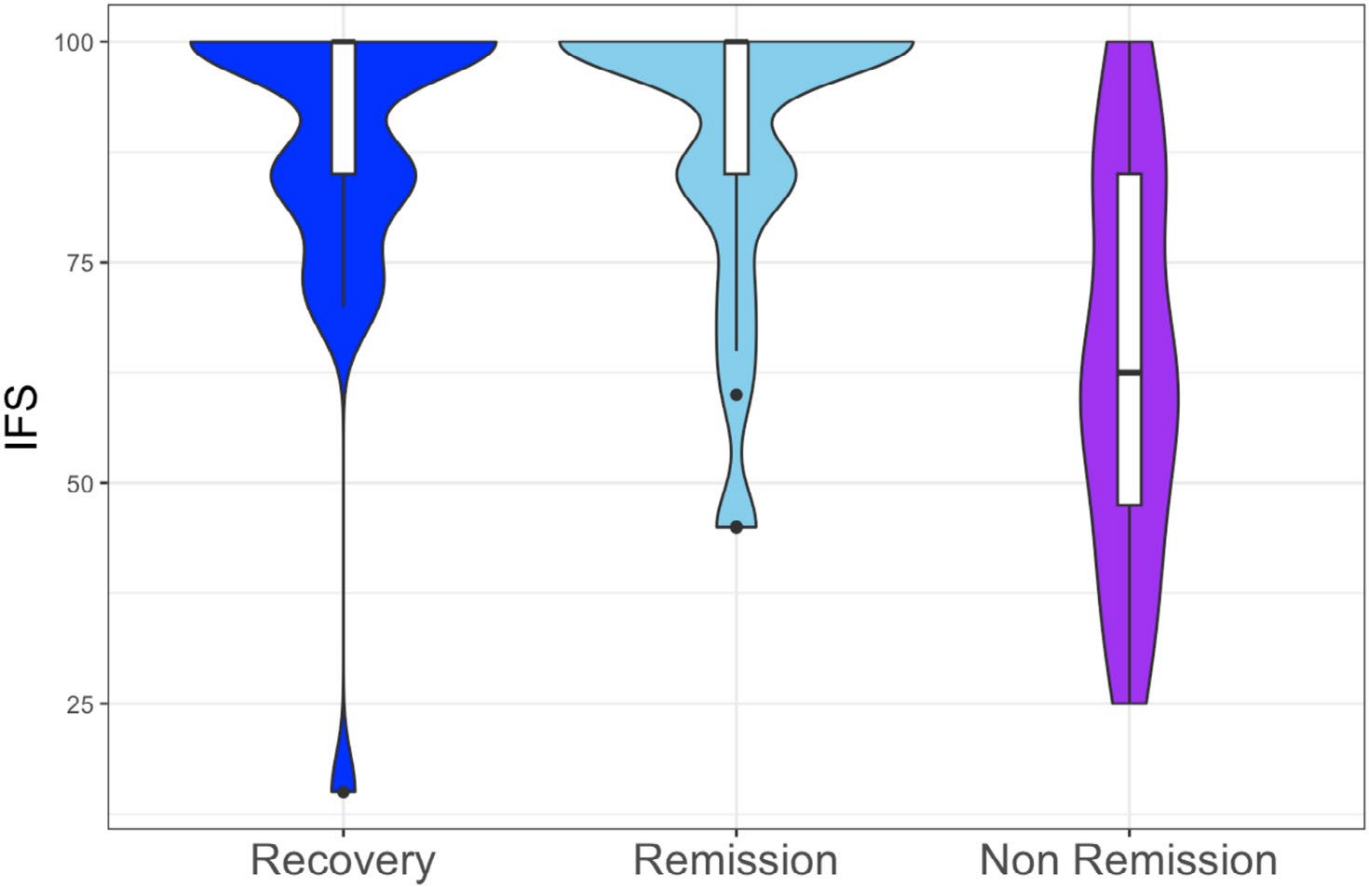
The comparison of IFS among recovery, remission, and non‐remission groups. IFS: Individual fitness score.

Figure 1 presents the comparison of IFS between the three groups, showing significantly higher IFS in recovery and remission groups than in the non‐remission group (*p* < 0.01). In the post hoc test, the IFS was significantly higher in the recovery group than in the non‐remission group (*p* < 0.01), and the IFS of the remission group was also significantly higher than that of the non‐remission group (*p* < 0.01). Conversely, no significant difference was observed in the IFS between the recovery and remission groups (*p* = 0.86).

### 3.3 Correlation Between IFS and Clinical Characteristics

Table 3 shows the correlation between IFS and demographic or clinical characteristics across the groups. No significant correlation was observed, except for the age of onset in the recovery group.

**TABLE 3:** Correlation between IFS and clinical characteristics among each schizophrenia group.

	Recovery		Remission		Non‐remission	
	*r*	*p*	*r*	*p*	*r*	*p*
Age (year)	−0.155	0.471	−0.070	0.712	−0.168	0.506
BMI	−0.305	0.147	0.129	0.497	−0.238	0.341
Education (year)	−0.023	0.914	0.117	0.537	−0.234	0.350
Duration of employment (year)	−0.369	0.076	−0.022	0.909	0.441	0.067
Duration of illness (year)	0.017	0.937	−0.092	0.628	−0.371	0.129
Average daily dosage (CPZ‐equivalent, mg/day)	−0.131	0.543	−0.011	0.954	−0.049	0.846
Number of admissions	0.011	0.961	−0.157	0.407	−0.211	0.401
Age at onset (year)	−0.572	0.004	−0.030	0.875	0.180	0.475
GAF	−0.027	0.901	0.102	0.593	0.397	0.115
BPRS	−0.195	0.361	−0.094	0.623	0.194	0.441
CDSS	0.067	0.754	−0.176	0.351	0.152	0.547

Abbreviations: BPRS, Brief Psychiatric Rating Scale; CDSS, Calgary Depression Scale for Schizophrenia; CPZ‐ equivalent, Chlorpromazine equivalent, GAF, Global Functioning Assessment.

### 3.4 ROC Analysis of Remission and Non‐Remission Group

**FIGURE 2:**
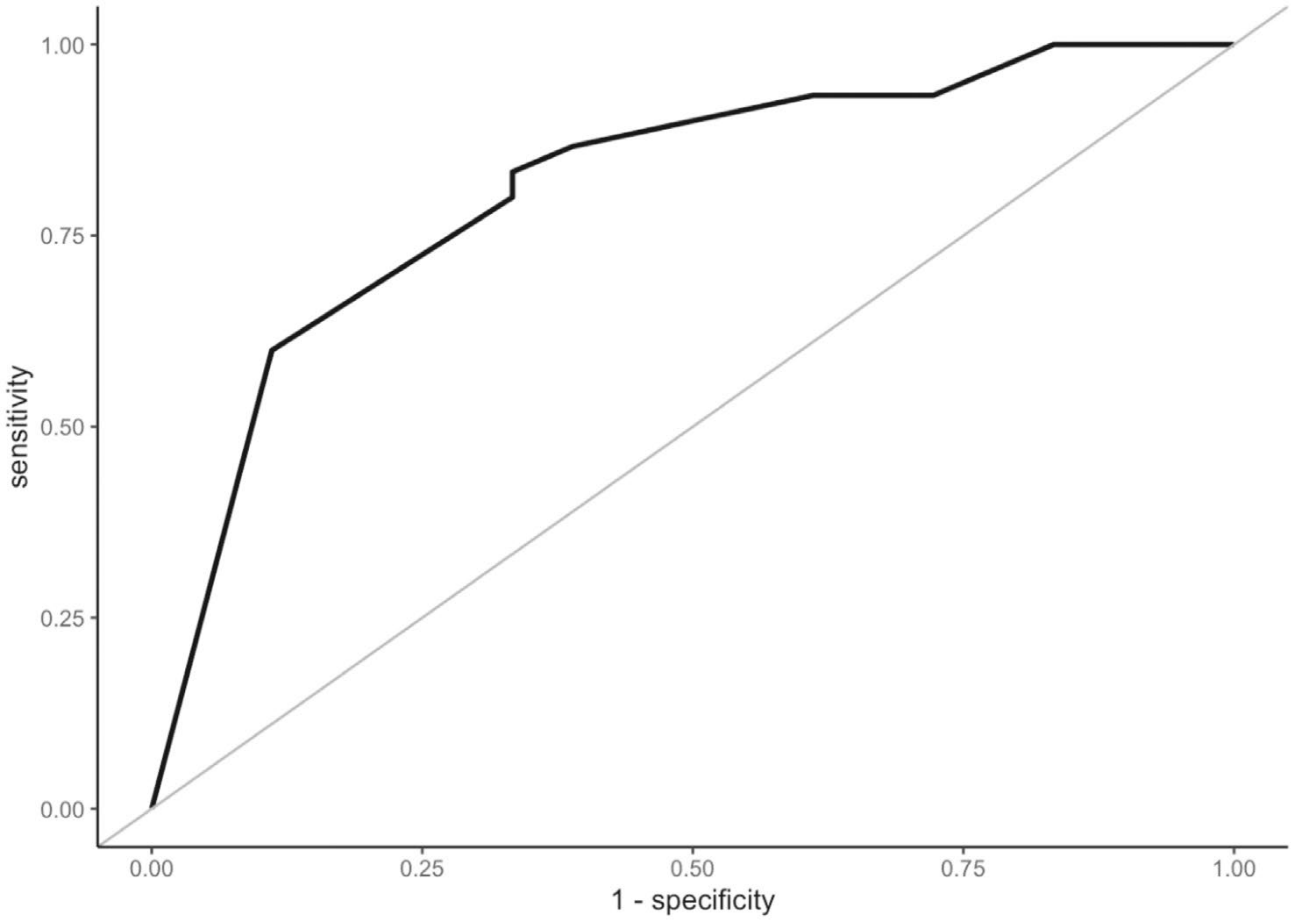
ROC curve for cut off between remission and non‐remission schizophrenia group.

Based on the ROC curve, an IFS cutoff point of 72 showed 83.3% sensitivity and 67.67% specificity, with an area under the curve of 0.814 [95% CI: 0.686–0.942] (Figure 2). The optimal cutoff point was determined using the Youden index.

## 4 Discussion

This is the first study to evaluate the association between guideline adherence and the state of patients with schizophrenia. We found that the IFS in patients with schizophrenia in recovery and remission was significantly higher than that in patients with schizophrenia in non‐remission. In addition, an IFS cutoff of 72 points may help distinguish between remission and non‐remission pharmacotherapy. However, no significant difference in IFS was observed between the recovery and remission groups.

In recovery and remission patients with schizophrenia, adherence to guidelines was higher than that in non‐remission patients; however, no significant difference in IFS was observed between the recovery and remission groups. This suggests that adherence to the guidelines may contribute to achieving remission in patients with schizophrenia. This study suggests that adherence to the guidelines may be effective, as indicated in previous studies [7, 8]. However, this study uniquely evaluated the different states of schizophrenia, including recovery, remission, and non‐remission. The meta‐analysis surveying the global trend of prescription showed that the prevalence of antipsychotic polypharmacy was higher in Asia than North America and Oceania, and the concomitant use of antiepileptics or anticholinergics in antipsychotics was higher in Asia than in other regions [17, 18]. Other studies comparing the prescriptions in 16 Asian countries reported that the Japanese prevalence of antipsychotics polypharmacy and concomitant use of psychotropics was higher than in other countries [19]. We considered that the number of antipsychotics or concomitant use of psychotropics might have a greater influence on the difference in IFS than the dose of antipsychotics, as the CPZ equivalent was < 600 mg/day across the three groups in the present study. In post hoc analysis, the dose of CPZ equivalent was not a significant difference between the remission and non‐remission groups. This result indicated that non‐remitted patients with schizophrenia might not receive an adequate dose of antipsychotic treatment and might receive inadequate doses of antipsychotics polypharmacy or combination treatment with antipsychotics and other psychotropics. Furthermore, the result of ROC analysis showed an IFS of 72 points, but this result was obtained from an exploratory analysis, and it was difficult to conclude to distinguish remission and non‐remission schizophrenia. Several guidelines have outlined the appropriate dose and duration of antipsychotics [3, 4]; however, no indicator exists for the overall prescription for each patient. This result may provide a clinical target for achieving remission in non‐remission patients with schizophrenia.

No significant difference in IFS was observed between the recovery and remission states. Our findings suggested that adherence to the guidelines was not sufficient to achieve recovery, which may indicate limitations in the guidelines for pharmacological therapy. The IFS of recovery and remission were higher than those in previous reports comparing [7, 8], and we considered that adherence to the guidelines remained important. Conversely, the results of this study may indicate that other factors than drug therapy, such as psychosocial factors, are important in achieving recovery from remission. In fact, several psychological interventions, such as cognitive remediation, psychoeducation, social skills training, and cognitive behavioral therapy, were useful for improving social functions and preventing relapse [20].

This study indicated that the IFS has two aspects in evaluating pharmacotherapy, serving as a comprehensive evaluation tool for prescriptions and therapeutic indicators. First, the JSNP guideline includes some recommendations regarding the number of medications, dose of antipsychotics, and concomitant use of psychotropics [5], and IFS could evaluate these quality indicators (QIs) simultaneously. The difference in IFS may indicate a comprehensive adherence to these QIs, and we considered that adherence to multiple QIs may contribute to achieving remission in patients with schizophrenia. However, the IFS of the recovery and remission groups also suggest that complete adherence to the guidelines may not necessarily contribute to achieving recovery or remission in patients with schizophrenia. In fact, some studies have reported the efficacy of antipsychotic polypharmacy for positive symptoms or the prevention of rehospitalization [21, 22], and combining antipsychotics and antidepressants improved negative symptoms in patients with schizophrenia [23]. While adherence to treatment guidelines is generally beneficial, some clinical situations may require individualized modifications, such as dose adjustments or selective polypharmacy, to achieve optimal outcomes. In addition, this study was designed to include the patients taking no anticholinergic drugs, compared to previous studies [7, 8]. While the calculation of IFS included the anticholinergic drugs, the Japanese prescription survey showed that the prescription of anticholinergic drugs was correlated with high‐dose antipsychotics prescription, and was also influenced depend on the institutions [24]. To minimize the influence between institution on IFS score, we included only the schizophrenia patients taking no anticholinergic drugs in this study. Second, the IFS has the possibility as a clinical indicator for pharmacotherapy. The exploratory ROC analysis may suggest the possibility of a score which distinguishes between remission and non‐remission. It is difficult to conclude a causal relationship between IFS score and treatment response in schizophrenia in this study. Only a few biomarkers are associated with remission in patients with schizophrenia [25]. However, in the future, if various IFS cutoff points depended on the state are known in large sample study, an appropriate pharmacotherapy targeting IFS for each patient with schizophrenia may be proposed. Thus, the IFS may serve not only as a tool for evaluating adherence to guidelines but also as a clinical target for pharmacotherapy across different states in patients with schizophrenia.

This study has some limitations. First, the cross‐sectional study design could not reveal causality between the IFS and recovery, remission, and non‐remission states; longitudinal studies are needed to assess causality. Second, the sample size was relatively small, and the IFS cutoff point which was obtained from exploratory analysis should be interpreted with caution. The results from this study should be evaluated in a larger‐scale study. Third, lower IFS might be reflected in not only poor adherence to guideline but also severity of symptoms, more complex pharmacotherapy or individualized prescriptions. Fourth, the results of correlation between IFS and clinical characteristics were examined from multiple correlation and should be interpreted conservatively. Fifth, these results were influenced by the differences in education for psychiatrists, differences in treatment approach at each institution or the trend of psychotropic prescription because this study was conducted at five psychiatric institutions in Japan.

## 5 Conclusion

This study showed high adherence to guidelines in patients in recovery and remission states of schizophrenia, compared with those in non‐remission states, and no significant difference in IFS was observed between recovery and remission. In addition, the cutoff point of IFS obtained from exploratory analysis may suggest that IFS could serve as a clinical indicator. This study showed that adherence to guidelines is important for recovery and remission; however, psychological factors may be crucial for achieving recovery from remission. We believe that assessing IFS in long‐term individual studies with large‐scale samples is necessary.

## Author Contributions

R.A. designed the study, recruited participants, performed analysis, analyzed data, wrote the original draft of the manuscript, and provided funding. H.I. reviewed the manuscript. L.G. reviewed the manuscript. K.Y. recruited participants. H.H. designed the study, recruited participants, analyzed data, and reviewed the manuscript. All authors have read and approved the final manuscript.

## Funding

This work was supported by the Grant of The Clinical Research Promotion Foundation, 2022.

## Ethics Statement

This study was approved by the Fukuoka University Medical Ethics Review Board (approval number: U21‐11‐018).

## Consent

All patients provided written informed consent before participation in this study.

## Conflicts of Interest

The authors declare no conflicts of interest.

## Data Availability

The data that support the findings of this study are available on request from the corresponding author. The data are not publicly available due to privacy or ethical restrictions.
